# Effects of rhetorical devices on audience responses with online videos: An augmented elaboration likelihood model

**DOI:** 10.1371/journal.pone.0282663

**Published:** 2023-03-16

**Authors:** Guangchao Charles Feng, Yiwen Luo, Zhenwei Yu, Jinlang Wen

**Affiliations:** 1 Department of Interactive Media, School of Communication, Hong Kong Baptist University, Hong Kong, China; 2 College of Communication, Shenzhen University, Shenzhen, Guangdong, China; 3 College of Liberal Arts, Jiangxi Normal University, Nanchang City, Jiangxi, 330022, China; Bialystok University of Technology: Politechnika Bialostocka, POLAND

## Abstract

The way in which information is linguistically presented can impact audience attention, emotion, and cognitive responses, even if the content remains unchanged. The present study aims to examine the effects of rhetorical devices on audience responses by introducing a new theoretical framework, the augmented elaboration likelihood model (A-ELM), which integrates elements of the Elaboration Likelihood Model and narrative theory. The results show that the mediation effects of attention on the relationships between rhetorical devices and affective and cognitive elaborations are moderated by involvement. Nonnarrative evidence, combined narrative and numerical evidence, source credibility, and tropes *versus* the lack of figures of speech, elicit better audience responses in low-involvement situations, whereas numerical evidence outperforms narratives in high-involvement situations. This study not only offers a novel theoretical framework in the form of A-ELM, but also has important implications for advancing methodologies and practical applications.

## Introduction

The manner in which information is linguistically presented has a profound impact on how audiences attend to, process, and respond to it emotionally, even when the content remains the same [1]. This is particularly relevant in today’s digital world, where online videos have become a prevalent form of media consumption. These videos are not only used for entertainment purposes, but they also serve as sources of information dissemination, product advertising, and political campaigning [2]. Therefore, it is crucial to understand the role of narratives and rhetorical strategies in shaping audience responses to online videos, particularly given the widespread influence of these videos on society.

Despite numerous studies exploring the persuasive effects of linguistic styles, there is still a gap in our understanding of how rhetorical strategies interact to influence audience responses in real-world settings. Most prior studies have relied on small-scale empirical studies to examine the effects of rhetorical figures on various outcomes, such as attention, ad attitudes, brand attitudes, purchase intention, preference, and memorability [3–7]. These studies have often ignored the potential interaction between rhetorical figures and other message features, such as narratives.

To fill this gap, the present study aims to investigate the effects of rhetorical devices and the possible interactions among them on audience responses to online videos. By combining big data mining and content analysis methods, this study seeks to provide a more comprehensive understanding of the relationship between narratives and rhetorical devices and audience responses. The study also proposes an integrated framework that incorporates theories from information processing and narratives, which has the potential to address the inconsistent and mixed findings of prior studies.

This new study is necessary as it has the potential to inform the development of more effective communication strategies and deepen our understanding of how language shapes the way in which we attend to, process, and respond to information, particularly in the context of online videos. With the growing influence of online videos, a better understanding of the interplay between message strategies and audience responses has the potential to lead to more impactful and effective communication.

### Audience responses

Prior research has demonstrated that the use of rhetorical figures in messages can enhance audience engagement by eliciting responses in attention, cognitive (i.e., thoughts), and affective (i.e., feelings) elaborations [8,9]. These responses are susceptible to different message features and heuristic cues, and rhetorical devices serve as relevant influencers. For instance, Weber [10] discovered that less factual news is conducive to audiences’ perceived relevance and engagement among many news (message) factors. This finding alludes to the positive effect of narratives and other rhetorical devices on audience responses, each of which will be reviewed below.

Thoroughly examining the relationships among attentive (e.g., viewing or the first time clicking), cognitive (e.g., commenting), and affective (e.g., liking) responses is beyond the scope of the present study. Nevertheless, it is widely accepted that attention acts as a gatekeeper for both cognitive and affective responses [11–14]. This is especially relevant in the context of online videos, where users must click on the video to view it before they can engage with it through likes, shares, and comments. The relationship between cognition and affect, however, remains a topic of intense debate [15–17]. Rather than delving into this debate, this study considers affective and cognitive elaborations as parallel response variables of attention.

Consequently, the following hypothesis is proposed:

H1: Attention to the headline is positively associated with subsequent affective (e.g., liking) (H1a) and cognitive elaborations [i.e., nonverbal (e.g., sharing) and verbal (e.g., commenting) elaborations (H1b)].

In what follows, various rhetorical devices, particularly narratives and figures of speech, and their effects on audience responses are reviewed, and hypotheses and research questions are raised accordingly.

### Narratives

#### Definitions

Scholars from different disciplines have provided varying definitions of the term “narrative”. It is considered a symbolic representation of events [18,19]. [20] expanded the definition of narratives to include stories with plots and chronological sequences of events [19,21]. Although “narrative” often appears as a generic term in the literature, it can encompass various forms, such as personal stories, anecdotes, eyewitness accounts, entertainment-education content, and testimonials [20,22].

#### Narrative effects

Previous research has consistently demonstrated the persuasive effects of narratives. The underlying mechanism is that once individuals become immersed in the story, there is a transportation effect that causes people to perceive the story as realistic and identify with its characters [23]. According to [23], individuals who are transported into a narrative world are likely to change their real-world beliefs through reduced negative cognitive responses and counterarguments, the realism of the experience, and strong affective responses [also see 24]. These findings are echoed by the extended elaboration likelihood model [24], the entertainment overcoming resistance model (EORM) [25], and exemplification theory [26].

While many studies have confirmed the persuasive effect of narrative content, some scholars have challenged these findings. Some have found a nonsignificant [27] or even reversed [28] effect of narratives *versus* nonnarratives on attitude changes or knowledge and perception. In addition [29–32], have found that a particular form of nonnarrative, i.e., numerical evidence, is more persuasive than narrative evidence. [33] contended that numerical evidence has a stronger effect than narrative evidence on beliefs and attitudes, whereas narrative evidence has a stronger influence on intentions. Follow-up studies by [29,34] found that a message combining narrative and numerical evidence is more persuasive than using either form of evidence alone. This finding was also replicated by [35].

#### Moderation effect of involvement on the relationship between narratives and responses

[36] attributed mixed findings regarding the persuasive effects of narratives and numerical evidence to the influence of involvement. [36] also found that narrative testimonials are more compelling than numerical evidence under low involvement conditions. indicating that the effect of narrative is a heuristic process [36]. [37] suggested that individuals who are highly engaged in a narrative should have low levels of involvement and ability in scrutinizing a persuasive argument, while those highly involved in a compelling argument should concentrate on the quality of claims.

A series of related hypotheses are raised below:

H2: The effects of other forms of nonnarrative evidence, numerical evidence, and combined narrative and numerical evidence on attention are stronger than those of narrative evidence depending on the level of involvement. Specifically, narrative evidence receives greater attention than other forms of nonnarrative evidence (H2a), numerical evidence (H2b), and combined narrative and numerical evidence (H2c) under low involvement conditions.

H3: The indirect effects of other forms of nonnarrative evidence, numerical evidence, and combined narrative and numerical evidence on liking are stronger than those of narrative evidence depending on the level of involvement. Specifically, narrative evidence receives higher levels of liking than other forms of nonnarrative evidence (H3a), numerical evidence (H3b), and combined narrative and numerical evidence (H3c) under low involvement conditions.

H4: Similar to H3, narrative evidence receives higher levels of cognitive elaboration than other forms of nonnarrative evidence (H4a), numerical evidence (H4b), and combined narrative and numerical evidence (H4c) under low involvement conditions.

### Figures of speech

#### Conceptualization

A figure of speech (FoS), defined as “a form of speech artfully varied from common usage” [38], is a vital message style that affects persuasive effects [39]. Although over two hundred different figures have been cataloged, many are variants of approximately forty general types of figures of speech [40] [also cf. [41]]. These figures of speech can be divided into two major groups, i.e., tropes and schemes [38]. Tropes, including specific figures such as metaphors, puns, and associations, involve the substitution or destabilization of the meaning of a word that is a deviation from what it usually signifies. Schemes, which are characterized by the reversal of word order, omissions, insertions, repetitions, and rhyme, deviate from customary grammatical structure [5,38,40]. In the same vein, [42] classified rhetorical figures into two types, namely, metabolas and parataxis, which correspond to tropes and schemes, respectively.

The persuasive effects of rhetorical figures have been examined in various contexts and found to be effective, for example, advertisements that use rhetorical figures are more likely to garner greater attention [3], ad attitudes, brand attitudes, purchase intention [4], preference, and memorability [5–7].

#### Moderation effect of involvement on the relationship between FoS and responses

The persuasive effects of a figure of speech (FoS) have also been found to be moderated by involvement. In studying a specific type of FoS, i.e., rhetorical questions, [43] found that the effect of rhetorical questions on message attention and elaboration is stronger at levels of low involvement. The reason for this is that rhetorical questions can distract message recipients from processing arguments and can also make recipients perceive pressure from the source [44]. A similar finding has been observed in a few ad copy studies [7,45,46]. Consequently, a series of related hypotheses are proposed below:

H5: Tropes (H5a) and schemes (H5b) receive greater attention than literal texts that do not use any figures of speech under low involvement conditions.

H6: The mediation effects of attention on the relationships between figures of speech and affective elaboration (liking) are moderated by involvement such that tropes (H6a) and schemes (H6b) receive higher levels of liking than literal texts that do not use figures of speech under low involvement conditions.

H7: The mediation effects of attention on the relationships between figures of speech and cognitive elaboration are moderated by involvement such that tropes (H7a) and schemes (H7b) receive higher elaboration than literal texts that do not use any figures of speech under low involvement conditions.

### Source credibility

#### Conceptualization

As reviewed above, the credibility of information sources [for a review of the conceptualization and operationalization, see 47] affects the effect of persuasion [48]. Previous studies have found that highly credible (trustworthy and competent) sources produce a more positive attitude toward advocacy than do sources that are perceived to be less credible [for a review, see [49]].

#### Moderation effect of involvement on the relationship between source credibility and responses

The persuasive effect of source credibility is moderated by involvement [also cf. [50,51]]. When people have little involvement in an issue, a source with low credibility will induce a more positive attitude than will a more credible communicator [52]. According to the dual-process theories reviewed above, source credibility belongs to one of the peripheral cues that people rely on mainly under low involvement conditions in information processing [53,54] [for a review, see [55]]. That is, a highly credible source has a positive persuasive effect only when the level of involvement is low.

The following series of related hypotheses are raised accordingly:

H8: The effect of source credibility on attention is higher under low involvement conditions.

H9: The mediation effect of attention on the relationships between source credibility and liking is moderated by involvement such that higher levels of source credibility receives a higher level of liking under low involvement conditions.

H10: The mediation effect of attention on the relationships between source credibility and cognitive elaboration is moderated by involvement such that the higher level of source credibility receives higher levels of cognitive elaboration under low involvement conditions.

### Augmented ELM (A-ELM)

The kind of processing strategy that a message recipient employs depends on the number of mental resources the recipient is motivated to allocate to such information processing [56]. Moreover, due to the varied mental resources (involvement, ability, and opportunity), the recipient will follow two relatively distinct routes in persuasion [57]. This theorizing is well documented in dual-process models, including the elaboration likelihood model (ELM) [57,58], the heuristics-systematic model (HSM) [59], the motivation, opportunity, and ability model (MOA) [60], and others.

The extended elaboration likelihood model (E-ELM) [24,61] hypothesizes that the processing process and outcome of entertainment content are determined by one’s engagement with the story plot and characters. Authors have argued that only involvement with the characters rather than issue involvement is relevant in such a context [24]. Nevertheless, such theorizing assumes that all entertainment content is homogeneous in substance, forms, and styles. [62] proposed the language complexity (simple vs. complex) × processing mode (automatic vs. controlled) framework (LCPMF), which considers the abovementioned variations of content. However, the relationship between language complexity and processing mode is entangled, and the LCPMF has never been empirically applied. In addition, language complexity may not fully account for the variation in processing strategies. For instance, a message recipient who is emotionally engaged with the story plot and characters or finds the message entertaining may maintain a constant processing mode, regardless of its level of complexity.

**Fig 1:**
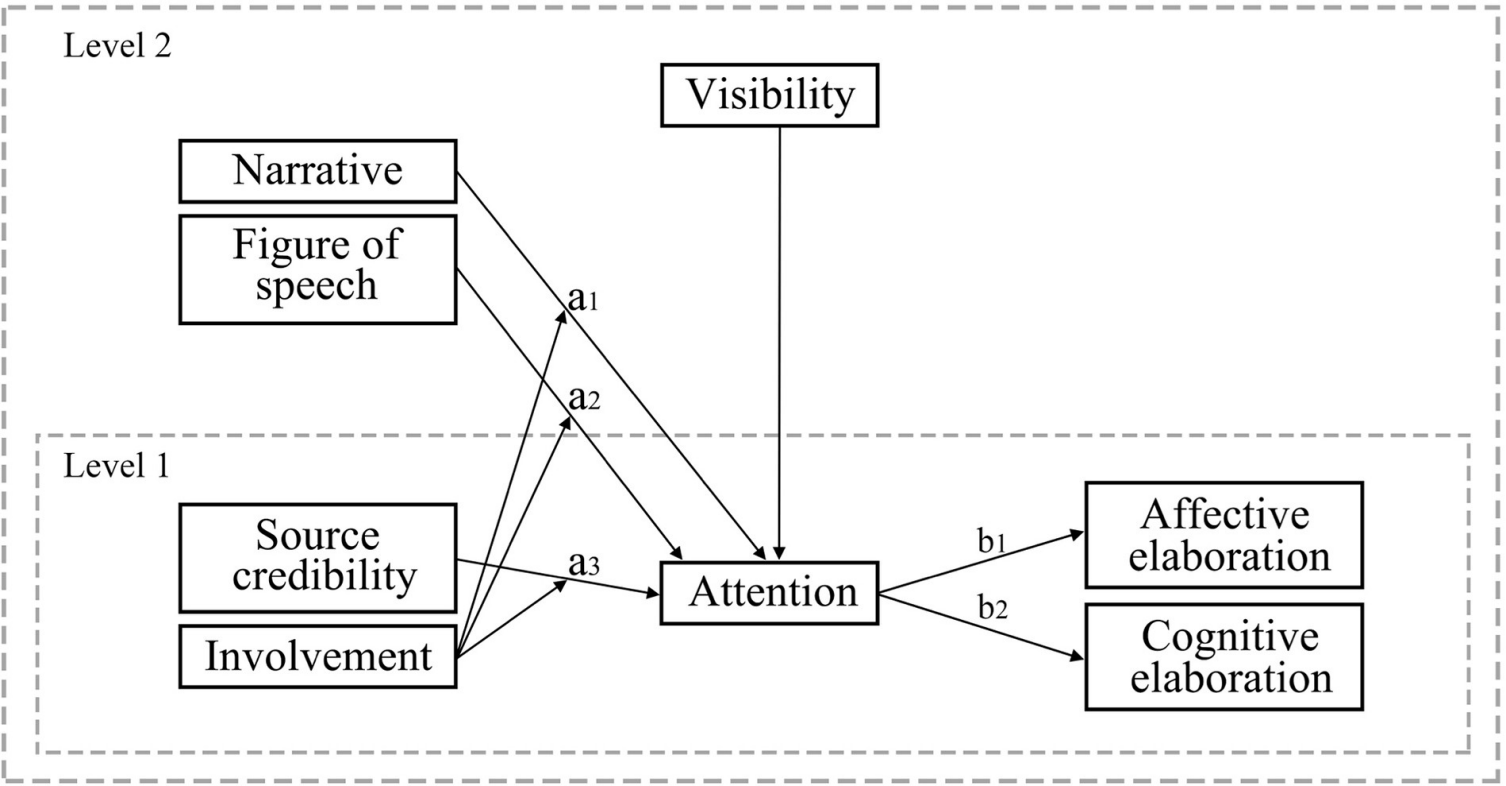
Multilevel moderated mediation model.

Moreover, the LCPMF fails to consider any cues other than language complexity, such as language intensity [63], narrative or numerical evidence, or the credibility of content creators, which might affect the processing strategies adopted. The present study proposes a new integrated theoretical framework called the augmented ELM (A-ELM) by incorporating narrative theory and the ELM. The A-ELM, as shown in Fig 1, comprises all the hypotheses and research questions raised above. In summary, it maintains that the mediation effects of attention (click to view) on the relationships between rhetorical devices and elaborations are moderated by issue involvement.

## Method

### Procedure

#### Data mining

Most data were collected from two major video-sharing websites in China, namely, Xigua Video (2,648,807 items or 35% of the total) and Bilibili (4,919,998 items or 65% of the total), through data scraping using a crawler programmed with Python. The elements of data to be scraped include the title of the video and the audience responses received for the content, which include the number of views, the number of likes, the number of saves, the number of shares, the number of danmaku (Danmu or bullet chats), and the number of comments, among others. In addition, the attribute information, including the visibility of the issue covered in the text and the author’s impact, e.g., the number of followers, was collected.

#### Content analysis and codebook

After the data had been collected from the websites, ten thousand randomly sampled items were subjected to further content analysis (9,244 were left after removing those titles with fewer than five characters). The tests addressing the hypotheses and research questions were based on the coding results of the content analysis. Five senior-year undergraduate and four postgraduate students majoring in the Chinese language were recruited to code the sampled data according to the codebook.

The codebook describes and explains the categories of each variable, which will work as the independent variables used in later hypothesis testing. The coded independent variables included figures of speech [three categories including 1) tropes, 2) schemes, and 3) none] and types of evidence [four categories including 1) narrative, 2) numerical, 3) the combination of the two, and 4) other Nonnarrative evidence].

Each of the coders was trained by one of the three research assistants (RAs) and allowed to start formal coding only after their coding results had satisfactory intercoder reliability with those of a corresponding RA. The RAs also randomly chose samples of coding results from each coder to check their coding quality every day. If intercoder reliability was a problem, the coder of concern was asked to recode certain content.

The intercoder reliability test using the frequently used indices showed that all the categories’ intercoder reliability was satisfactory [64–67]. In addition to the results of the intercoder reliability test, the tables are also presented in the online appendix (https://doi.org/10.6084/m9.figshare.19498751.v1) due to the space limit.

### Measures

#### Audience responses to headlines

As discussed above [8,9], audience responses to headlines include paying attention to the headline (viewing) and subsequent affective (e.g., liking the story) and cognitive (e.g., commenting) elaborations after watching the video. These metrics were collected by scraping the relevant websites.

#### Viewing

Viewing was measured by the number of viewers (*M* = 547,654, *SD* = 1,056,431, *N* = 9,244).

#### Affective Elaboration

Affective elaboration, or liking, was measured by the number of likes (*M* = 26,521, *SD* = 65,718, *N* = 8,182).

#### Cognitive Elaboration

Cognitive elaboration was measured by the number of shares (*M* = 2155, *SD* = 8,308, *N* = 8,168), the number of saves (*M* = 6,311, *SD* = 24,119, *N* = 8,168), the number of comments (*M* = 692, *SD* = 1,825, *N* = 9,242), and the number of Danmaku (overlaying comments on video displays) (*M* = 3,398, *SD* = 8,747, *N* = 5,022).

Principal component analysis (PCA) with varimax rotation was performed to examine the scale’s dimensionality based on the big data (*N =* 7,568,805). The number of extracted factors in the PCA was determined based on the eigenvalue-greater-than-one rule [68]. The PCA yielded two factors (verbal and nonverbal cognitive elaborations) that explained 85% of the variance in the items. The factor loadings of verbal elaboration were .86 (comments) and .90 (Danmaku), and the Cronbach’s α was .72. The factor loadings of nonverbal elaboration were .94 (shares) and .95 (saves), and the Cronbach’s α was .87. Given the acceptable performance on validity and reliability, the dimensionality results derived from PCA were used in the following structural equation modeling (SEM).

### Independent variables

#### Types of Evidence

The four categories of evidence were coded as narrative (70.857%), numerical (.876%), the combination of the two (1.309%), and other nonnarrative evidence (26.958%).

#### Figures of Speech

There were three included categories of figures of speech, i.e., tropes (18.714%), schemes (12.166%), and none (69.120%).

#### Source Credibility

Source credibility was measured by the number of followers of content creators (*M* = 1,025,659, *SD* = 3,386,276, *N* = 9,244) and the fame of content creators.

#### The Number of Followers

The number of followers a content creator has is often used as a peripheral cue by consumers to gauge the creator’s trustworthiness and credibility [69,70]. Moreover, [69] discovered a positive effect of the number of followers a celebrity has on the subject’s perception of the celebrity’s attractiveness, trustworthiness, and competence [cf. 71]. This information was collected by scraping the existing data of the websites.

#### The Fame of Content Creators

The authors of videos come from a variety of sources, including 1) traditional media, 2) new media platforms, 3) official organizations, 4) nongovernmental organizations (NGOs), 5) celebrities, 6) ordinary anchors (equivalent to YouTubers), and 7) famous anchors. A cluster analysis was performed to rank the content creators by the degree of fame. The result showed three ordered categories of fame, i.e., 6, 2 and 7 combined, and the rest combined.

The same PCA procedures as those discussed above were performed on the two items based on the big data (*N =* 7,568,805). The PCA yielded one factor that explained 79% of the variance in the items. The factor loadings were .8 and .88, and the Cronbach’s α was .865.

#### Issue Involvement

Involvement can indicate the importance of an issue and the amount of needed effort to process information [72] [for a taxonomy of involvement, see [73]]. The study’s data sources could be a natural proxy measure of involvement due to the difference in the needed effort of browsing. The two video platforms have very diverse user bases. The users of Bilibili are mostly generation Z and fans of ACGN (animations, comics, games, and novels) [74,75], while the users of Xigua are, in general, generally older people who care about serious topics [76,77]. Therefore, the users of Bilibili who aim to seek stimulation have a shorter attention span and are less likely to pay attention to the details in the text of titles [cf. 78] than those of Xigua. In light of the fundamental difference in the user characteristics of the two platforms, the level of involvement was measured by the sources of the videos. Bilibili (Bilibili = 2, 31.556%) and Xigua (Xigua = 1, 68.444%) indicate conditions of high and low involvement, respectively.

#### Control Variables

There were two control variables that served as control variables, i.e., topic visibility and the date difference between publishing and crawling times.

#### Topic Visibility

Topic visibility or familiarity, which might affect attention, worked as a control variable [cf. 79]. The visibility of the concerning event or people, as often displayed in social media metrics, indicates the object’s popularity [80]. This visibility also serves as a heuristic cue for the algorithms and the users of video websites alike to perceive that object as valuable and noteworthy [81]. This variable must be controlled because the trending topic easily dominates the attention of users, and people are hence less likely to be affected by the rhetorical strategies contained in the title that has recently been widely discussed on social media.

The visibility of the concerned topic was measured by the number of search times obtained by scraping the “hot searches” provided by Weibo, which is a Twitter-like website in China. The video titles are matched with the “hot searches” database during the same period. Those that were successfully matched received a rating score of visibility, whereas those that failed to match were left blank (NA) (*M* = 812,146, *SD* = 1,062,156, *N* = 8,499).

#### Date Differences

Videos published in earlier days will have more extended viewing periods and, hence, a better chance to be viewed (*M* = 171, *SD* = 294, *N* = 8,473). Controlling for the effect of this date difference aimed to rule out the influence resulting from the artifact.

#### Model specification

As stated in the hypotheses and RQs above, attention mediates the effects of rhetorical devices on affective and cognitive elaborations. Moreover, such a mediation process is moderated by the level of involvement, which forms the moderated mediation model [for a review of this method, see [82]]. In light of [82], the significant prediction of either *a*_*j*_ (the prediction from predictor *X* to mediator *M*) or *b*_*j*_ (the prediction from *M* to dependent variable *Y*) depending on moderator *W* indicates the presence of moderated mediation. The predictions of *a*_*j*_ depending on *W* (involvement) have been included in H5 and H6, while the predictions of *b*_*j*_ are not dependent on involvement due to a lack of theoretical support.

The proposed framework is tested using a 2-1-1 (level 2 predictors—level 1 mediator–level 1 outcome) multilevel moderated mediation model. The model specification is explained as follows:

$${Like}_{ij}=d_{Yj}+{b1}_{j}*{ATT}_{ij}+e_{Y_{ij}},$$ 1

$${ATT}_{ij}=d_{Mj}+{a1}_{j}*INVO+e_{M_{ij}},$$ 2

$${a1}_{j}=\gamma_{1}+\gamma_{2}*{IVs}_{j}+e_{j},$$ 3

where the subscripts *i* and *j* indicate the individual observations (responses metrics) and platform IDs, respectively. *X*_*j*_*s* are level-2 predictors (IVs), including the linguistic features, source credibility, and control variables. The mediator (*M*_*ij*_) is level-1 attention (ATT), and the moderator is level-1 involvement (INVO). Furthermore, *a*1_*j*_ denotes the random effect of involvement on attention and is predicted by the level-2 factors. Consequently, *γ*_2_ is the cross-level interaction effect, i.e., the moderation effect of involvement on attention. In summary, the model predicts that the level-2 linguistic characteristics predict affective and cognitive elaborations (the outcome variable at level 1) via attention (mediator at level 1). Moreover, this mediation effect is moderated by involvement at level 1.

The indirect effects of the linguistic factors on affective and cognitive elaborations are the products of the effects of *a* (the effects of linguistic factors on attention) and *b* (the effects of attention on affective and cognitive elaborations). The expected value of one of the indirect effects of linguistic factors conditional on involvement can be obtained as follows:

$$E(a_{j}b_{j})|{(INVO}_{ij})=(\gamma_{2}*{b1}_{j}*{INVO}_{ij}).$$ 4

### Dataset and analysis

The data were scraped, cleaned, and manipulated using Python 3.7. The dataset is stored on the online repository (https://doi.org/10.6084/m9.figshare.19498751.v1). Some analyses (e.g., PCA and reliability test) and data manipulation also employed R 3.6, and hypothesis testing using structural equation modeling (SEM) was performed with M*plus* 8.6 [83]. The collection and analysis method complied with the terms and conditions for the source of the data.

## Results and discussions

### Results

The intraclass correlation coefficient (ICC) derived from the mixed-effects null model was .295, indicating that 29.5% of the variance was accounted for by clustering. As a result, multilevel modeling was necessary. The hypotheses and research questions were addressed using multilevel moderated mediation modeling with SEM and the Monte Carlo and numerical integration method, which. However, this method made *χ*^2^ and related fit statistics unavailable.

All the measurement models were significant. The number of shares and saves were significantly loaded on the factor of nonverbal cognitive elaboration, whereas the number of comments and danmaku were significantly loaded on the factor of verbal cognitive elaboration. Moreover, the two factors were significantly loaded on a single factor, which is simplified as cognitive elaboration. In addition, the number of followers and the fame of content creators were significantly loaded on a single factor, i.e., source credibility.

Both affective elaboration (i.e., the number of likes) and cognitive elaboration were significantly predicted by attention (i.e., the number of viewers) (*β* = .713, *p*<.001; *β* = .423, *p*<.001). That is, higher levels of attention translate into higher levels of affective and cognitive elaborations. Consequently, H1 was supported.

Depending on the level of involvement, the effects of other forms of nonnarratives (vs. narratives) and combined narrative and numerical evidence on attention were significant (*β* = .043, *p*<.01; *β* = .176, *p*<.01. That is, nonnarrative evidence and combined narrative and numerical evidence attract more viewers than narrative evidence under low involvement conditions. Numerical evidence attracts more viewers under high involvement conditions, as expected, but this effect was insignificant (*β* = −.111, *p* = .179). Consequently, H2a and H2c were supported, but H2b was rejected.

Regarding the effect of figures of speech (FoS) on attention, only the effect of tropes (vs. nonuse of FoS) was significant (*β* = .061, *p*<.01). That is, tropes can attract more viewers than literal texts under low involvement conditions. Nevertheless, there was no significant difference in attracting viewers between schemes and the nonuse of FoS. Consequently, H5a was supported, but H5b was rejected.

The effect of source credibility on attention was significant (*β* = 32.211, *p*<.001). That is, the higher the source credibility is, the more viewers there are when the involvement level is low. Therefore, H8 was supported.

The effect of the control variables (i.e., visibility of the issue and date difference) on attention was also significant (*β* = −.062, *p*<.001; *β* = .116, *p*<.001). Higher levels of visibility attract fewer viewers under low involvement conditions. The longer that videos are published, the more viewers they have. In summary, controlling for the effects of visibility and date difference, the effects of numerical and other forms of nonnarrative evidence, tropes, and source credibility on attention were significant depending on the varying level of involvement.

The moderated mediation effects of attention on the relationships between rhetorical devices (and source credibility) and affective elaboration (e.g., liking), and cognitive elaboration had mixed results. The effects of the combination of narrative and numerical evidence, other forms of nonnarrative (vs. narrative) evidence, tropes (vs. nonuse of figure of speech), and source credibility on both affective and cognitive elaborations were significantly mediated by attention depending on the level of involvement. There were significant moderated mediation effects of the combination of narrative and numerical evidence, other forms of nonnarrative evidence, tropes and source credibility on liking, i.e., affective elaboration(*β* = .251, *p*<.01; *β* = .061, *p*<.01; *β* = .087, *p*<.001; *β* = 45.957, *p*<.001) and cognitive elaboration (*β* = .149, *p*<.01; *β* = .036,*p*<.01; *β*= .051, *p*<.05; *β* = 27.258, *p*<.001). Consequently, H3a, H3c, H4a, H4c, H6a, H7a, H9, and H10 were supported, but H3b, H4b, H6b, and H7b were rejected.

## Discussion

The relationships between the mediator (i.e., attention) and affective and cognitive elaborations were significant. Attention clearly plays the role of a gatekeeper for subsequent responses. Nevertheless, the effects of linguistic devices and source credibility on responses were not consistent.

There are significant and moderated (by involvement) mediation effects of attention on the relationships between the predictors [other forms of nonnarrative (vs. narrative) evidence, the combination of narrative and numerical evidence, tropes (vs. nonuse of figure of speech), and source credibility] and both affective and cognitive elaborations. When the level of involvement was low, the combination of narrative and numerical evidence, other forms of nonnarrative evidence, tropes, and source credibility had positive effects on audience responses. In contrast, visibility had positive effects on responses under high involvement conditions. The positive effects of other forms of nonnarrative evidence and the combination of narrative and numerical evidence under low involvement conditions could be attributed to two factors.

First, in the context of video viewing, the titles using narratives have already included a synopsis of their stories. If users have low issue involvement, then they lack the motivation to click to watch the video. Second, an untested three-way interaction may exist on top of the moderation of involvement. For instance, the genre of videos, which is an attribute not reported by Xigua Video, may moderate the interaction effect of involvement and evidence types. In addition, older age groups are more easily attracted by narratives, while members of generation Z [75], who comprise the staple user base of Bilibili, prefer nonnarratives with or without numbers over narratives in their browsing [cf. 84].

## Research implications

This study has made substantial theoretical contributions by proposing an augmented ELM (A-ELM) that combines narrative theory and dual-process theories into a unified theoretical framework. The A-ELM represents an improvement over previous ELM(s) in multiple ways.

The A-ELM is different from the E-ELM [24,61], which holds that the audience’s emotional engagement (transportation) is only relevant to high levels of involvement with the plots and characters. Involvement with the plots and characters is different from issue involvement. The former, which is a core concept in the E-ELM, should be better conceptualized as narrative engagement, while the latter is simply involvement that the ELM refers to. Moreover, the E-ELM contends that communication outcomes are determined by involvement with the story plot and characters, while the A-ELM maintains that mere narrative evidence is detrimental to communication outcomes when issue involvement is low. The underlying reason is that users lack the motivation to process the information further when they are not interested in the issue per se.

The A-ELM provides a fresh perspective on the debate over the persuasive impact of narratives versus numerical evidence. Nonnarratives should be differentiated between numerical and nonnumerical ones. Numerical evidence indicating a higher argument quality works better under high involvement conditions. In contrast, other nonnarrative evidence is believed to have better audience responses under low involvement conditions.

In addition, tropes and source credibility have better effects on audience responses under the low involvement condition, while video titles that address social issues tend to attract users with high levels of involvement.

The present study employed two methods, i.e., big data mining and content analysis. The validity and reliability of the measurement scales were first confirmed based on big data collected from the natural setting, and then the hypotheses and research questions were examined based on randomly selected data subjected to content analysis. In addition, the study adopted innovative multilevel moderated mediation modeling based on Monte Carlo integration. Therefore, this study has made several methodological contributions to the field.

This study not only has theoretical and methodological implications, but also practical implications for video creators. Depending on the users that a video targets, video creators can choose specific rhetorical devices to grab attention. Specifically, creators should use nonnarrative evidence and figures such as metaphors, puns, and other similar tropes in video titles to engage young users who lack persistent levels of involvement. Nevertheless, video titles with numerical evidence and public issues work best to engage older users who are interested in serious topics.

## Conclusions

This study found that attention (number of viewers) is a significant predictor of affective (number of likes) and cognitive (e.g., number of comments) elaborations. Higher levels of attention result in higher levels of affective and cognitive elaborations. This study also found that nonnarrative evidence and combined narrative and numerical evidence attract more viewers than narrative evidence under low involvement. The use of tropes in figures of speech was found to attract more viewers than literal texts under low involvement. It implies that when reading texts with tropes and non-narratives, users apparently process those messages effortlessly as peripheral cues. Users, however, process numerical information, particularly concerning trending social issues, in an effortful and systematic way.

Source credibility was found to have a significant effect on attention, with higher credibility leading to more viewers under low involvement. The control variables, i.e., visibility of the issue and date difference, were also found to have a significant effect on attention.

## Limitations

The mixed results of the study could be attributed to several limitations. Firstly, the study only utilized data from two major video-sharing websites in China, which may have resulted in the exclusion of other important sources and websites. Secondly, the impact of narratives on communication could be influenced by transportation and narrative involvement, but the extent of narrative involvement was not accessible to the study. Finally, due to the use of secondary data, many constructs were measured using a limited number of items, leading to concerns about reliability.

## Future research directions

Based on the limitations mentioned above, future research directions in this area could include:

1. To gain a more comprehensive understanding of the communication effects of rhetorical devices and narratives, the scope of data sources could be expanded to include additional video-sharing websites, such as those in China and beyond, including YouTube [cf. 2].
2. Enhancing Measurement Reliability. To address the reliability concerns in the measurement of constructs, future research could utilize multiple items or more robust measures. Furthermore, while the primary users of the two video-sharing platforms were originally comprised of Generation Z and older adults, these platforms are now evolving to attract a more diverse range of age groups. Therefore, the measurement of involvement could be improved by using additional variables such as content focus (e.g. political vs entertainment).
3. Investigating the effects of rhetorical devices and narratives in different contexts. The effects of rhetorical devices and narratives may vary across different communication contexts, such as in politics, entertainment, advertising, or health. Future research could examine the effects in these different contexts and compare the results to the findings of this study.
4. Conducting qualitative studies to complement the quantitative findings, and explore the underlying mechanisms and processes that explain the communication effects of rhetorical devices and narratives.

These future research directions could help to further our understanding of the effects of rhetorical devices and narratives on communication outcomes and inform the development of more effective communication strategies that use rhetorical devices and narratives.
